# Radiographical assessment of post and core placement errors encountered by Saudi dental students at different educational levels

**DOI:** 10.12688/f1000research.137421.1

**Published:** 2023-08-14

**Authors:** Turki Alshehri, Nourhan M. Aly, Raand Altayyar, Deena Alghamdi, Shahad Alotaibi, Passent Ellakany

**Affiliations:** 1 College of Dentistry, Imam Abdulrahman Bin Faisal University, Dammam, Eastern Province, Saudi Arabia; 2 Department of Pediatric Dentistry and Dental Public Health, Faculty of Dentistry, Alexandria University, Alexandria, Egypt; 3 Department of Substitutive Dental Sciences, College of Dentistry, Imam Abdulrahman Bin Faisal University, Dammam, Saudi Arabia

**Keywords:** Dental students, Post and Core, Radiograph, Gutta Percha, Endodontically treated.

## Abstract

**Background:** Dental post and core is one of the common procedures performed after endodontic treatment. The aim of this study was to radiographically assess the quality of post and core procedures performed by dental students at different education levels in addition to determining the most critical errors encountered during their clinical practice.

**Methods:** A retrospective cross-sectional study design was conducted in the College of Dentistry, Imam Abdulrahman Bin Faisal University. A total of 550 periapical radiographs (PAs) of cemented posts were retrieved from the records of patients treated by dental students. Parameters and guidelines for assessing the quality of post treatment have been determined and statistically analyzed. A P value <0.05 was considered statistically significant.

**Results:** The study included 502 students and most of them were females (66.5%). Data were obtained from 502 patients (62% females) with fiber posts used in 98.2% of the cases. About 50% of the posts were inserted in premolars, 62.9% in the upper arch, and 66.7% were restored with crowns as a final restoration. Regarding the quality of posts, 98.4% showed good preparation quality and 98% showed good radiographic quality. The post diameter was equal to 1/3 of the root diameter in 31.9% of the cases; post length was equal to 2/3 of root length in 5% of the cases and equal to or more than crown height in all cases (100%). Length of the remaining gutta percha (GP) was between 3–5 mm in 38.8%, and there was no gap between the post and remaining GP in 95.6% of the cases. There were no statistically significant differences between dental students at different clinical educational levels regarding the quality of post placement.

**Conclusions:** The quality of post and core procedures performed by students showed acceptable radiographic quality and were within the recommended standards.

## Introduction

Restoration of endodontically treated teeth (ETT) with extensive coronal tooth loss requires the placement of dental posts and core that adds retentive features to the coronal restoration. ^1^ Several post systems were developed either in the form of custom-made posts using gold or non-precious metals, or prefabricated stainless steel or titanium posts. ^2^ Aesthetic posts were recently introduced as alternative options including ceramic and glass fiber posts with variable shapes and sizes. ^3^

Success of post and core procedures depends on following the proper sequence of the treatment plan in addition to the accuracy of each step performed prior to post placement. ^4^ One of the reliable methods used for evaluating the post placement procedure is taking periapical radiographs (PAs) prior to, during and after post cementation. ^5^ Several factors affect the longevity of dental posts such as proper filling and obturation, acceptable apical seal, and absence of clinical and radiographic signs and symptoms. ^6^

Dental students are required to perform several clinical procedures as a prerequisite for graduation. One of these procedures is post and core placement in ETT followed by a final prosthetic restoration. ^4^^,^^7^ Students perform post and core procedures in their clinical years following well-designed rubrics for each procedure under the supervision of their faculty supervisors to achieve the optimum outcome in each step of the treatment plan. ^4^^,^^5^ Accordingly, these rubrics provide proper assessment of the students’ practice and skills level, and also helps in improving their ability to perform self-assessment for each specific dental procedure.

The evaluation criteria of the post and core quality include coronal tooth preparation, radicular canal preparation and post-operative cementation of the fiber post. Several studies illustrated the capability of undergraduate dental students in post placement in ETT among Saudi Universities. ^4^^,^^7^^,^^8^ However, no studies were conducted to assess the students’ abilities and skills in post and core placement in the Eastern Province region. Hence, the aim of this study was to radiographically assess the quality of post and core performed by dental students at different educational levels in addition to determining the most critical errors encountered during their clinical practice. The null hypothesis states that there would be no statistically significant differences in the performance of dental students at different educational levels.

## Methods

A retrospective cross-sectional study was conducted in the College of Dentistry, Imam Abdulrahman Bin Faisal University. A total of 550 periapical radiographs (PAs) of cemented fiber posts were collected and retrieved from the records of patients treated by dental students at Imam Abdulrahman bin Faisal University Dental Hospital. The sample size was estimated assuming 80% study power and 5% alpha error. ^9^ The study was approved by the institution research board (IRB-2022-02-285) of Imam bin Abdulrahman bin Faisal university, dammam, Saudi Arabia.

**Figure 1.**
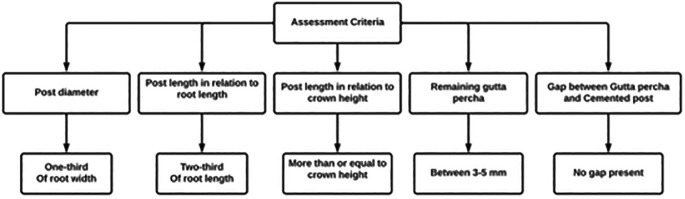
Criteria of post and core evaluation.

All PAs of prefabricated posts cemented on ETT performed by undergraduate dental students in the fourth, fifth, sixth and internship years from 2018 till 2022 were included. These PAs were assessed according to the following criteria by four trained investigators ( Figure 1). ^4^^,^^8^^,^^10^

Pre-operative and post-operative PAs were assessed to confirm the tooth status before post placement and to determine if any errors resulted from the post placement. Poor-quality PAs, those with missing treatment details, or missing postoperative PAs were excluded. Medicor Imaging Picture Archiving and Communication System (Weasis is a free medical DICOM viewer) Dental Enterprise Viewer was used to visualize the PAs using patients’ IDs. Dental arch, tooth number, and the type of post (metal or fiber) were recorded in an excel sheet. Moreover, the assessment of the quality of canal preparation (acceptable: outline of the preparation was following the canal contour and unacceptable: canal preparation did not follow the canal outline or ledge resulted during preparation) and the quality of PAs were added to the excel sheet where the followed evaluation criteria of PAs were extracted from a previous study criteria. ^11^ The length of the placed post was measured starting from the beginning of the restoration to the apical tip of the post as well as the crown height and root length which were measured from the cementoenamel junction to the root apex. Additionally, the length of the remaining gutta-percha in the canal, the root width and post width at the middle half of the root, and the gap between the post and gutta-percha if available, were it was measured using a digital ruler of the viewer software. Any abnormalities noticed in the PAs were also recorded in the sheet.

### Statistical analysis

Descriptive data were calculated as frequencies, percentages, means and standard deviations (SD). Comparisons between the three study groups were done using chi-square test for qualitative variables, and one-way ANOVA for quantitative variables. Data were analyzed using IBM SPSS for Windows (Version 23.0). P value <0.05 was considered statistically significant. An alternative proprietary free suggested software is ocscsistatistics.

## Results

The study included 502 students (348 fifth and sixth year students, 101 fourth year students and 53 interns) and most of them were females (66.5%). Data were obtained from 502 patients (62% females) with fiber posts used in 98.2% of the cases ( Table 1). Table 2 shows the different parameters of included posts. About 50% of the posts were inserted in premolars, 62.9% in the upper arch, and 66.7% restored with crowns as a final restoration. Regarding the quality of posts, about 98% showed good preparation and radiographic quality. As for post assessment according to the ideal prosthetic criteria ( Table 3); the post diameter was equal to 1/3 of the root diameter in 31.9% of the cases, post length was equal to 2/3 of root length in 5% of the cases, post length was equal to or more than crown height in all cases (100%), the length of the remaining GP was between 3–5 mm in 38.8%, and there was no gap between the post and remaining GP in 95.6%. There were no statistically significant differences between dental students at different clinical educational years regarding the quality of post placement according to the criteria mentioned in the methods.

**Table 1.** Demographic characteristics of the study sample (n=502).

Variable	Categories	Program level				χ ^2^ P value
		4 ^th^ (n=101)	5 ^th^ and 6 ^th^ (n=348)	Interns (n=53)	Total (n=502)	
		N (%)				
**Patient gender**	**Male**	53 (52.5%)	124 (35.6%)	14 (26.4%)	191 (38%)	0.002 *
	**Female**	48 (47.5%)	224 (64.4%)	39 (73.6%)	311 (62%)	
**Student gender**	**Male**	42 (41.6%)	103 (29.6%)	23 (43.4%)	168 (33.5%)	0.02 *
	**Female**	59 (58.4%)	245 (70.4%)	30 (56.6%)	334 (66.5%)	

* Statistically significant at p value <0.05.

**Table 2.** Evaluation parameters of the included posts (n=502).

Variable	Categories	Program level				χ ^2^ P value
		4 ^th^	5 ^th^ and 6 ^th^	Interns	Total	
		N (%)				
**Arch**	**Maxillary**	71 (70.3%)	212 (60.9%)	33 (62.3%)	316 (62.9%)	0.23
	**Mandibular**	30 (29.7%)	136 (39.1%)	20 (37.7%)	186 (37.1%)	
**Type of tooth**	**Anterior**	28 (27.7%)	95 (27.3%)	18 (34%)	141 (28.1%)	0.61
	**Premolar**	51 (50.5%)	180 (51.7%)	21 (39.6%)	252 (50.2%)	
	**Molar**	22 (21.8%)	73 (21%)	14 (26.4%)	109 (21.7%)	
**Type of post**	**Fiber**	99 (98%)	342 (98.3%)	52 (98.1%)	493 (98.2%)	0.98
	**Metal**	2 (2%)	6 (1.7%)	1 (1.9%)	9 (1.7%)	
**Quality of preparation**	**Good**	99 (98%)	342 (98.3%)	53 (100%)	494 (98.4%)	0.61
	**Poor**	2 (2%)	6 (1.7%)	0 (0%)	8 (1.6%)	
**Quality of radiographs (PA)**	**Good**	99 (98%)	342 (98.3%)	52 (98.1%)	492 (98%)	0.73
	**Poor**	2 (2%)	6 (1.7%)	1 (1.9%)	10 (2%)	
**Length of post**	**Mean (SD)**	15.67 (2.46)	15.80 (2.86)	15.97 (3.08)	15.79 (2.80)	0.82
**Remaining GP**	**Mean (SD)**	5.92 (1.81)	6.33 (1.80)	5.77 (1.36)	6.19 (1.77)	0.10
**Gap between post and GP**	**Yes**	7 (6.9%)	12 (3.4%)	3 (5.7%)	22 (4.4%)	0.29
	**No**	94 (93.1%)	336 (96.6%)	50 (94.3%)	480 (95.6%)	
**Type of final restoration**	**Crown**	53 (52.5%)	249 (71.6%)	33 (62.3%)	335 (66.7%)	0.001*
	**Composite**	48 (47.5%)	99 (28.4%)	20 (37.7%)	167 (33.3%)	
**Radiographic abnormalities**	**Yes**	0 (0%)	0 (0%)	0 (0%)	0 (0%)	1.00
	**No**	101 (100%)	348 (100%)	53 (100%)	502 (100%)	

**Table 3.** Posts’ assessment according to the ideal prosthetic criteria.

Variable	Categories	Program level				χ ^2^ P value
		4 ^th^	5 ^th^ and 6 ^th^	Interns	Total	
		N (%)				
**Post diameter**	**Less than 1/3 root width**	70 (69.3%)	218 (62.6%)	30 (56.6%)	318 (63.3%)	0.17
	**Equals 1/3 root width**	25 (24.8%)	117 (33.6%)	18 (34%)	160 (31.9%)	
	**More than 1/3 root width**	6 (5.9%)	13 (3.7%)	5 (9.4%)	24 (4.8%)	
**Post length**	**Less than 2/3 root length**	5 (5%)	6 (1.7%)	1 (1.9%)	12 (2.4%)	0.27
	**Equals 2/3 root length**	4 (4%)	20 (5.7%)	1 (1.9%)	25 (5%)	
	**More than 2/3 root length**	92 (91.1%)	322 (92.5%)	51 (96.2%)	465 (92.6%)	
**Remaining GP length**	**Less than 3 mm**	0 (0%)	1 (0.3%)	0 (0%)	1 (0.2%)	0.12
	**Between 3-5 mm**	49 (48.5%)	122 (35.1%)	24 (45.3%)	195 (38.8%)	
	**More than 5 mm**	52 (51.5%)	225 (64.7%)	29 (54.7%)	306 (61%)	

One-way ANOVA was used.

## Discussion

Success and longevity of post and core restorations depend on several criteria such as post preparation dimensions in relation to tooth dimensions, post length relative to tooth length in addition to material of the used post. All these criteria can be radiographically evaluated, thus, the current study aimed to radiographically assess the quality of post and core procedures performed by dental students at different education levels in addition to determining the most critical errors encountered during their clinical practice.

The study findings showed that 98.4% of the students did post preparations of acceptable quality. According to the criteria mentioned in the methodology, about 31.9% of students placed posts of diameter equivalent to 1/3 of the root diameter, while only 5% of the students used posts of length equal to 2/3 of root length in their cases. The acceptable length of the remaining GP (3–5 mm) was reported in 38.8% of the cases. Nonetheless, no gap was noticed between the posts and remaining GP in 95.6% of the cases which ensures optimum adaptation of the posts cemented by the students. The quality of students’ performance in post placement was acceptable and comparable among different clinical educational years. Thus, the null hypothesis was accepted.

Results of the current study showed that 63.3% of the posts were cemented in maxillary teeth which comes in line with previous studies. ^4^^,^^8^^,^^12^^–^^15^ Ease of isolation, absence of saliva and absence of tongue movements that can obscure vision and affect the quality of post restoration in the maxillary arch might be a valid explanation for this percentage. ^16^ In case of type of the teeth restored, students commonly treated premolars followed by anterior teeth, while molars were less frequently treated. Similarly, a previous study reported that premolars were the most restored teeth with post and cores by undergraduate dental students. ^8^ Another study showed that incisors were the most frequently restored teeth with posts followed by premolars. ^4^ Selection of teeth by students might be related to the ease of post preparation and placement, so most students prefer treating single rooted teeth as several complications can be encountered while treating multi-rooted teeth including perforations and root fractures. ^17^

Metal posts were placed in nine cases only, while fiber posts were cemented in 493 cases. The high esthetic demand of patients was the cause of this difference where metal post can affect the shade of the definitive restoration. Also, placement of fiber posts provides better adhesion to the tooth through resin cements and reduces the percentage of vertical root fractures that might result from torquing the serrated metal posts. ^18^^–^^20^

Most of the restored teeth (95.6%) showed no gap between the cemented post and GP. This comes in agreement with Almaghrabi *et al.* 14 who stated that about 93% of cases did not show any gaps. Also, Mathar *et al.* ^8^ reported that 82.9% of cases showed no gaps between the cemented post and GP. In evaluating the post length in relation to root length, 92.6% of the cases were following the optimum guidelines of exceeding 2/3 of the root length. Similarly, Almaghrabi *et al.* ^15^ showed that post length was less than 2/3 of the root length in 61% of cases. However, Meshni *et al.* ^4^ found that the post to root length ratio in almost half of the patients was 2:1.

The study findings showed that almost 31% of the cases were treated with post diameter equivalent to 1/3 of the root diameter. In line with these findings, previous studies reported that the diameter of the cemented posts was equivalent to 1/3 of the root. ^21^^,^^22^ Additionally, another study conducted at Qassim University dental clinics showed that 81% of the post cases were of length equal to 1/3 of the root. ^8^ This optimum post diameter dimensions agrees with Trabert *et al.* ^23^ who recommended a post diameter not exceeding 1/3 of the root width to increase fracture resistance of the restoration ^22^ in addition to reducing the possibility of root fracture. ^24^

The ideal amount of the remaining GP in the root after post preparation ranges between 3-5 mm, and this was found in 38.8% of the cases. This comes in agreement with Al Maghrabi *et al.* ^15^ who used the same criteria in their cases. They reported 3-5 mm remaining GP in 68% of cases done in King Abdulaziz university. ^15^ Also, a different study performed in Jazan University found that 55.7% of the assessed cases were within the same range of remaining GP. ^4^

The high percentages of cases following the standard guidelines of post and core placement and cementation among variable teeth (incisors, premolars and molars) might be justified by the presence of Faculty supervision with each student in the educational clinics to limit the complications or errors that can happen in the patient’s mouth. Additionally, following explicit calibrated rubrics by students and Faculty members in placing post and core restorations and assessing their quality might have positively affected the treatment outcome. Inclusion of different students of various dental educational levels presents generalized findings on the performance of students in the dental college of Imam Abdurrahman University. This can improve the dental curriculum of undergraduate students in order to enhance their knowledge and psychomotor skills.

However, the current study had some limitations such as being restricted to Eastern province so the findings cannot be generalized to the broader dental population in Saudi Arabia. Also, it included undergraduate students with the exclusion of postgraduate students since the postgraduate endodontic and prosthodontics boards were recently introduced in the dental college and the total number of enrolled students do not exceed ten students. Further studies are needed to compare the performance of undergraduate and postgraduate students. Moreover, assessment of post and core quality needs to be extended in other Saudi dental colleges to detect the weaknesses in students’ performance.

## Conclusions

Dental students showed acceptable performance in placing post and cores in ETT following the guidelines of post placement. No significant differences were reported between the performances of students of various educational years. Most of the students used narrow posts relative to the tooth diameter. Dental students’ fear of perforations of narrow canals or furcation perforations pushed them to treat mainly premolars and incisors and to use narrower posts than the proper diameter. Therefore, students need to have more confidence in their qualities to improve their clinical skills.

## Data Availability

Zenodo. Radiographical Assessment of Post and Core Placement Errors Encountered by Saudi Dental Students at Different Educational Levels, 10.5281/zenodo.8126789v. This project contains the following underlying data:
• Post data.xlsx (post and core data done by students). Data are available under the terms of the Creative Commons Attribution 4.0 International license (CC-BY 4.0).
