# Radiographic assessment of dental post and core placement at different educational levels in an undergraduate student clinic: a 4-year retrospective study

**DOI:** 10.12688/f1000research.137421.2

**Published:** 2024-03-28

**Authors:** Turki Alshehri, Nourhan M. Aly, Raand Altayyar, Deena Alghamdi, Shahad Alotaibi, Passent Ellakany

**Affiliations:** 1 College of Dentistry, Imam Abdulrahman Bin Faisal University, Dammam, Eastern Province, Saudi Arabia; 2 Department of Pediatric Dentistry and Dental Public Health, Faculty of Dentistry, Alexandria University, Alexandria, Egypt; 3 Department of Substitutive Dental Sciences, College of Dentistry, Imam Abdulrahman Bin Faisal University, Dammam, Saudi Arabia

**Keywords:** Dental students, Post and Core, Radiograph, Gutta Percha, Endodontically treated.

## Abstract

**Background:**

Dental post and core is one of the common procedures performed after endodontic treatment. The aim of this study was to radiographically assess the quality of post and core procedures performed by dental students at different education levels in addition to determining the most critical errors encountered during their clinical practice.

**Methods:**

A retrospective cross-sectional study design was conducted in the College of Dentistry, Imam Abdulrahman Bin Faisal University. A total of 550 periapical radiographs (PAs) of cemented posts were retrieved from the records of patients treated by dental students. Parameters and guidelines for assessing the quality of post treatment have been determined and statistically analyzed. A P value <0.05 was considered statistically significant.

**Results:**

The study included 502 students and most of them were females (66.5%). Data were obtained from 502 patients (62% females) with fiber posts used in 98.2% of the cases. About 50% of the posts were inserted in premolars, 62.9% in the upper arch, and 66.7% were restored with crowns as a final restoration. Regarding the quality of posts, 98.4% showed good preparation quality and 98% showed good radiographic quality. The post diameter was equal to 1/3 of the root diameter in 31.9% of the cases; post length was equal to 2/3 of root length in 5% of the cases and equal to or more than crown height in all cases (100%). Length of the remaining gutta percha (GP) was between 3–5 mm in 38.8%, and there was no gap between the post and remaining GP in 95.6% of the cases. There were no statistically significant differences between dental students at different clinical educational levels regarding the quality of post placement.

**Conclusions:**

The quality of post and core procedures performed by students showed acceptable radiographic quality and were within the recommended standards.

## Introduction

Endodontic treatment plays a major role in ensuring the survival of tooth and success of post and core treatment. ^1^ Restoration of endodontically treated teeth (ETT) with extensive coronal tooth loss requires the placement of dental posts and core that adds retentive features to the coronal restoration. ^2^ Several post systems were developed either in the form of custom-made posts using gold or non-precious metals, or prefabricated stainless steel or titanium posts. ^3^ Aesthetic posts were recently introduced as alternative options including ceramic and glass fiber posts with variable shapes and sizes. ^4^

Success of post and core procedures depends on following the proper sequence of the treatment plan in addition to the accuracy of each step performed prior to post placement. ^5^ One of the reliable methods used for evaluating the post placement procedure is taking periapical radiographs (PAs) prior to, during and after post cementation. ^6^ Several factors affect the longevity of dental posts, such as proper filling and obturation, acceptable apical seal, and absence of clinical and radiographic signs and symptoms. ^7^

Dental students are required to perform several clinical procedures as a prerequisite for graduation. One of these procedures is post and core placement in ETT followed by a final prosthetic restoration. ^5^^,^^8^ Students perform post and core procedures in their clinical years following well-designed rubrics for each procedure under the supervision of their faculty supervisors to achieve the optimum outcome in each step of the treatment plan. ^5^^,^^6^ Accordingly, these rubrics provide proper assessment of the students’ practice and skills level, and also helps in improving their ability to perform self-assessment for each specific dental procedure.

The evaluation criteria of the post and core quality include coronal tooth preparation, radicular canal preparation and post-operative cementation of the fiber post. Several studies illustrated the capability of undergraduate dental students in post placement in ETT among Saudi Universities. ^5^^,^^8^^,^^9^ However, no studies were conducted to assess the students’ abilities and skills in post and core placement in the Eastern Province region. The study was conducted to address the common errors encountered in post and core procedures performed by dental students. By recognizing these errors, faculty members can develop a curriculum that focuses on these specific issues and teaches students how to avoid or correct them. This will help prevent complications that may arise during these procedures. Additionally, exposing students to failure cases and teaching them how to handle clinical situations will enhance their knowledge and improve their performance. By learning from these experiences, students can develop better skills and become more proficient in their clinical practice. Hence, the aim of this study was to radiographically assess the quality of post and core performed by dental students at different educational levels in addition to determining the most critical errors encountered during their clinical practice. The null hypothesis states that there would be no statistically significant differences in the performance of dental students at different educational levels.

## Methods

A retrospective cross-sectional study was conducted in the College of Dentistry, Imam Abdulrahman Bin Faisal University. The sample size was estimated assuming 80% study power and 5% alpha error. ^10^ According to Muthar *et al.* ^11^ study, the acceptable sample size was 119 samples. The study was approved by the institution research board (IRB-2022-02-285) of Imam Abdulrahman Bin Faisal University, Dammam, Saudi Arabia. A total of 550 digital periapical radiographs (PAs) recorded using parallel technique were saved digitally on radiographic software (MiPACS Dental Enterprise Viewer 3.1.1404, Medicor Imaging, Charlotte, NC, Weasis is a free software available as a medical DICOM viewer) at the college of dentistry. ETT restored with cemented fiber posts were retrieved from the records of patients. The cases included in this study were those treated by dental students at Imam Abdulrahman bin Faisal University Dental Hospital from 2018 till 2022. Since this study was conducted retrospectively, patient consent was achieved before starting the treatment; as a step of the routine hospital procedures. The investigators maintained the anonymity of all extracted data, ensuring that patients' identities were kept undisclosed.

Inclusion criteria were set as follows: (1) dental students started clinical practice of endodontic treatments as well as prosthetic courses, starting from 4 ^th^ year till the 6 ^th^ year as well as students of internship program, (2) cemented post and core procedures on optimum ETT, (3) non-surgical root canal treatment (4) availability of complete radiographic records (a minimum of three good quality periapical radiographs: a preoperative, working length and postoperative radiographs). Exclusion criteria were set as follows: (1) permanent teeth with uncompleted endodontic treatment, (2) retreatment cases, (3) teeth previously treated surgically with apicectomy, and (4) dental records with missing or poor quality (non-diagnostic) radiographs.

**Figure 1.**
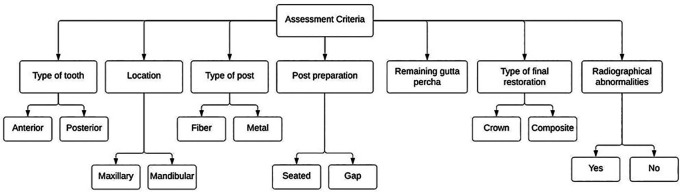
Assessment criteria of post and core treatment quality.

Medicor Imaging Picture Archiving and Communication System was used to evaluate PAs quality as either acceptable according to the absence of following items; cone cut, teeth overlap, elongation, or shortening or unacceptable. ^15^ Post and core treatments quality were also assessed in the extracted PAs by two trained investigators (S.A. and D.A) according to a previously published criteria ( Figure 1 and Table 1). ^5^^,^^9^^,^^11^ Before the assessment, the investigators underwent a calibration session to review the set criteria and analyze several post-obturation radiographs. The findings were discussed and an agreement was reached on the criteria of assessment. In this study, all clinical cases treated by students in the clinical sessions were approved and supervised by endodontic and prosthetic faculty member. The assigned clinical cases to the students were done by and endodontic faculty member where the cases were of low difficulty according to the American Association of Endodontics Case Difficulty Assessment Form and Guidelines. ^12^ Endodontic treatments were initiated by using either SS hand or engine-driven ProTaper Universal (PTU) files (Dentsply Maillefer, Ballaigues, Switzerland) followed by lateral condensation obturation technique using gutta percha cones and epoxy resin-based sealer (AH plus sealer, Dentsply Maillefer, Ballaigues, Switzerland). Post and core treatments were performed in the presence of rubber dam isolation according to the protocol of treatment in the dental school and under supervision of prosthodontic clinical staff where the average of staff to student ratio in the clinic was 1:8. However, the ratio in the internship was higher almost 1:10. The gutta percha was removed using a Gates drill (size 3) and a low-speed drill provided by the manufacturer of the post-system (glass fiber post; RelyX (3M ESPE) ^®^ Tapered post) Self-adhesive resin cement (RelyX U200/3M ESPE-U200; Maxcem Elite/Kerr-MAX; Clearfil SA Cement/Kuraray-CSA) was used to cement either the glass fiber or the metal posts (Dentatus Classic Surtex ^®^Posts, New York, USA) used. Dental arch, tooth number, and the type of post (metal or fiber) were recorded in an excel sheet. Moreover, the assessment of the quality of canal preparation (acceptable: outline of the preparation was following the canal contour and unacceptable: canal preparation did not follow the canal outline or ledge resulted during preparation) and the quality of PAs were added to the excel sheet where the followed evaluation criteria of PAs were extracted from a previous study criteria. ^5^^,^^9^^,^^11^^,^^13^ The length of the placed post was measured starting from the beginning of the restoration to the apical tip of the post as well as the crown height and root length which were measured from the cementoenamel junction to the root apex. Additionally, the length of the remaining gutta-percha in the canal, the root width and post width at the middle half of the root, and the gap between the post and gutta-percha if available, were it was measured using a digital ruler of the viewer software. Any abnormalities noticed in the PAs were also recorded in the sheet.

**Table 1.** Assessment guidelines of post and core treatment quality.

Assessment guidelines	Unacceptable (0)	Acceptable (1)	Ideal (2)
**Amount of remaining gutta percha**	1–3 mm	>5.1 mm	3.1–5 mm
**Gap between remaining gutta percha and post**	1.1–2 mm	0.1–1 mm	0 mm
**Post width to root width ratio**	>0.5	0.34–0.5	0.33
**Post length-to-root length ratio**	Reversed (0) 2/1	Minimal (1) 1/1	Optimal (2) 1/2 or 2/3
**Quality of radiographs**	Unacceptable (0) Cone cut, elongation, shortening, teeth overlap	Acceptable (1) Ideal dimensions of oral structures	
**Radiographic abnormalities**	Available (0)	Absent (1)	

### Statistical analysis

Descriptive data were calculated as frequencies, percentages, means and standard deviations (SD). Comparisons between the three study groups were done using chi-square test for qualitative variables, and one-way ANOVA for quantitative variables. Data were analyzed using IBM SPSS for Windows (Version 23.0). P value <0.05 was considered statistically significant. Alternative proprietary free suggested software is ocscsistatistics.

## Results

Data were obtained from 502 patients (62% females) with fiber posts used in 98.2% of the cases after excluding 48 cases due to the lack of complete data ( Table 2). About 62% of the treated cases were females. The cases assessed were treated by 348 fifth and sixth year students (69%), 101(20.1%) fourth year students and 53 (10.5%) interns. Most of the students were females (66.5%).

**Table 2.** Demographic characteristics of the study sample (n=502).

Variable	Categories	Program level				χ ^2^ P value
		4 ^th^ (n=101)	5 ^th^ and 6 ^th^ (n=348)	Interns (n=53)	Total (n=502)	
		N (%)				
**Patient gender**	**Male**	53 (52.5%)	124 (35.6%)	14 (26.4%)	191 (38%)	0.002 *
	**Female**	48 (47.5%)	224 (64.4%)	39 (73.6%)	311 (62%)	
**Student gender**	**Male**	42 (41.6%)	103 (29.6%)	23 (43.4%)	168 (33.5%)	0.02 *
	**Female**	59 (58.4%)	245 (70.4%)	30 (56.6%)	334 (66.5%)	

* Statistically significant at p value <0.05.

Table 3 shows the different parameters of included posts. About 50% of the posts were inserted in premolars, followed by anterior teeth and molars respectively (28.1% and 21.7%). Also, 62.9% of treated teeth were in the upper arch, and 66.7% restored with crowns as a final restoration while the rest of the cases were restored with composite restorations only. Regarding the quality of posts, about 98% showed good preparation and radiographic quality. The men value of post length was 15.79 mm where the longest posts were set by interns 15.97 while the shortest posts were seated by the 4 ^th^ year students 15.67 mm. The mean value of remaining GP in all treated cases was 6.19 mm with lowest reading among interns 5.77 mm followed by 4 ^th^ year 5.92 and 5 ^th^ and 6 ^th^ years 6.33 mm. No radiographic abnormalities were reported in all evaluated cases.

**Table 3.** Evaluation parameters of the included posts (n=502).

Variable	Categories	Program level				χ ^2^ P value
		4 ^th^	5 ^th^ and 6 ^th^	Interns	Total	
		N (%)				
**Arch**	**Maxillary**	71 (70.3%)	212 (60.9%)	33 (62.3%)	316 (62.9%)	0.23
	**Mandibular**	30 (29.7%)	136 (39.1%)	20 (37.7%)	186 (37.1%)	
**Type of tooth**	**Anterior**	28 (27.7%)	95 (27.3%)	18 (34%)	141 (28.1%)	0.61
	**Premolar**	51 (50.5%)	180 (51.7%)	21 (39.6%)	252 (50.2%)	
	**Molar**	22 (21.8%)	73 (21%)	14 (26.4%)	109 (21.7%)	
**Type of post**	**Fiber**	99 (98%)	342 (98.3%)	52 (98.1%)	493 (98.2%)	0.98
	**Metal**	2 (2%)	6 (1.7%)	1 (1.9%)	9 (1.7%)	
**Quality of preparation**	**Good**	99 (98%)	342 (98.3%)	53 (100%)	494 (98.4%)	0.61
	**Poor**	2 (2%)	6 (1.7%)	0 (0%)	8 (1.6%)	
**Quality of radiographs (PA)**	**Good**	99 (98%)	342 (98.3%)	52 (98.1%)	492 (98%)	0.73
	**Poor**	2 (2%)	6 (1.7%)	1 (1.9%)	10 (2%)	
**Length of post**	**Mean (SD)**	15.67 (2.46)	15.80 (2.86)	15.97 (3.08)	15.79 (2.80)	0.82
**Remaining GP**	**Mean (SD)**	5.92 (1.81)	6.33 (1.80)	5.77 (1.36)	6.19 (1.77)	0.10
**Gap between post and GP**	**Yes**	7 (6.9%)	12 (3.4%)	3 (5.7%)	22 (4.4%)	0.29
	**No**	94 (93.1%)	336 (96.6%)	50 (94.3%)	480 (95.6%)	
**Type of final restoration**	**Crown**	53 (52.5%)	249 (71.6%)	33 (62.3%)	335 (66.7%)	0.001*
	**Composite**	48 (47.5%)	99 (28.4%)	20 (37.7%)	167 (33.3%)	
**Radiographic abnormalities**	**Yes**	0 (0%)	0 (0%)	0 (0%)	0 (0%)	1.00
	**No**	101 (100%)	348 (100%)	53 (100%)	502 (100%)	

As for post assessment according to the prosthetic criteria ( Table 4); the post diameter was equal to 1/3 of the root diameter in 31.9% of the cases while 63.3% of the cemented posts were larger than 1/3 of root diameter. The post length was equal to 2/3 of root length in 5% of the cases but exceeding 2/3 of the root length in 92.6% of the cases. In case of evaluating the post length towards the crown height, all of the cemented posts were equal to or more than crown height (100%). The length of the remaining GP was between 3–5 mm in 38.8%, while 61% of the cases exceeded 5 mm of the GP remaining in the root. No gap was observed between the post and remaining GP in 95.6%. In assessing the students’ performance and clinical skills in treating ETT with post and core restorations, no statistically significant differences between dental students at different clinical educational years were noticed according to the criteria mentioned in the methods.

**Table 4.** Posts’ assessment according to the ideal prosthetic criteria.

Variable	Categories	Program level				χ ^2^ P value
		4 ^th^	5 ^th^ & 6 ^th^	Interns	Total	
		N (%)				
**Post diameter**	**Less than 1/3 root width**	70 (69.3%)	218 (62.6%)	30 (56.6%)	318 (63.3%)	0.17
	**Equals 1/3 root width**	25 (24.8%)	117 (33.6%)	18 (34%)	160 (31.9%)	
	**More than 1/3 root width**	6 (5.9%)	13 (3.7%)	5 (9.4%)	24 (4.8%)	
**Post length**	**Less than 2/3 root length**	5 (5%)	6 (1.7%)	1 (1.9%)	12 (2.4%)	0.27
	**Equals 2/3 root length**	4 (4%)	20 (5.7%)	1 (1.9%)	25 (5%)	
	**More than 2/3 root length**	92 (91.1%)	322 (92.5%)	51 (96.2%)	465 (92.6%)	
**Remaining GP length**	**Less than 3 mm**	0 (0%)	1 (0.3%)	0 (0%)	1 (0.2%)	0.12
	**Between 3-5 mm**	49 (48.5%)	122 (35.1%)	24 (45.3%)	195 (38.8%)	
	**More than 5 mm**	52 (51.5%)	225 (64.7%)	29 (54.7%)	306 (61%)	

One-way ANOVA was used.

## Discussion

Success and longevity of post and core restorations depend on several criteria such as post preparation dimensions in relation to tooth dimensions, post length relative to tooth length in addition to material of the used post. ^14^ All these criteria can be radiographically evaluated, thus, the current study aimed to radiographically assess the quality of post and core procedures performed by dental students at different education levels in addition to determining the most critical errors encountered during their clinical practice.

In the current dental school, 4 ^th^ year students start the clinical sessions of endodontic treatments and prosthodontics as well. However, in the 3 ^rd^ year, the students start the preclinical sessions of endodontics and prosthodontics. Students in all dental years are supervised by faculty member even in the internship year and all the treated cases are evaluated by the specialized faculty member for the initial approval to treat the case till finalizing the procedure required for the case. The student faculty ratio in preclinical and clinical years is 1:8, while in the internship the ration is 1:10.

The study findings showed that 98.4% of the students did post preparations of acceptable quality. According to the criteria mentioned in the methodology, about 31.9% of students placed posts of diameter equivalent to 1/3 of the root diameter, while only 5% of the students used posts of length equal to 2/3 of root length in their cases. The acceptable length of the remaining GP (3–5 mm) was reported in 38.8% of the cases. Nonetheless, no gap was noticed between the posts and remaining GP in 95.6% of the cases which ensures optimum adaptation of the posts cemented by the students. The quality of students’ performance in post placement was acceptable and comparable among different clinical educational years. Thus, the null hypothesis was accepted.

Results of the current study showed that 63.3% of the posts were cemented in maxillary teeth which comes in line with previous studies. ^5^^,^^9^^,^^15^^,^^16^ Ease of isolation, absence of saliva and absence of tongue movements that can obscure vision and affect the quality of post restoration in the maxillary arch might be a valid explanation for this percentage. ^17^ In case of type of the teeth restored, students commonly treated premolars followed by anterior teeth, while molars were less frequently treated. Similarly, previous studies reported that premolars were the most restored teeth with post and cores by undergraduate dental students. ^9^^,^^11^^,^^18^ Another study showed that incisors were the most frequently restored teeth with posts followed by premolars. ^5^ Selection of teeth by students might be related to the ease of post preparation and placement, so most students prefer treating single rooted teeth as several complications can be encountered while treating multi-rooted teeth including perforations and root fractures. ^19^

Metal posts were placed in nine cases only, while fiber posts were cemented in 493 cases. The high esthetic demand of patients was the cause of this difference where metal post can affect the shade of the definitive restoration. Also, placement of fiber posts provides better adhesion to the tooth through resin cements, comparable flexural strength, and modulus of elasticity to dentin and reduces the percentage of vertical root fractures that might result from torqueing the serrated metal posts. ^20^^–^^22^

Most of the restored teeth (95.6%) showed no gap between the cemented post and GP. This comes in agreement with Almaghrabi *et al*. ^16^ who stated that about 93% of cases did not show any gaps. Also, Mathar and Almutairi ^11^ reported that 82.9% of cases showed no gaps between the cemented post and GP. In contrast, Baik study ^18^ revealed that only 65% of treated cases had no gap between post and GP. The higher percentage in the current study might be due to the different treatment protocol or materials used. Also, the number of student faculty ratio might be a crucial factor that needs to be acknowledged in improving the percentage of success or survival rates. In evaluating the post length in relation to root length, 92.6% of the cases were following the optimum guidelines of exceeding 2/3 of the root length. Similarly, Almaghrabi *et al.* ^16^ showed that post length was less than 2/3 of the root length in 61% of cases. However, Meshini *et al.* ^5^ found that the post to root length ratio in almost half of the patients was 2:1.

The study findings showed that almost 31% of the cases were treated with post diameter equivalent to 1/3 of the root diameter. In line with these findings, previous studies reported that the diameter of the cemented posts was equivalent to 1/3 of the root. ^23^^,^^24^ Additionally, another study conducted at Qassim University dental clinics showed that 81% of the post cases were of length equal to 1/3 of the root. ^11^ This optimum post diameter dimensions agrees with Trabert *et al*. ^25^ who recommended a post diameter not exceeding 1/3 of the root width to increase fracture resistance of the restoration ^24^ in addition to reducing the possibility of root fracture. ^26^ This criteria was also shown in other studies where 89.5% and 81.6% the cases included posts of diameter 1/3 of the root diameter. ^5^^,^^18^ On the contrary, Peutzfeldt *et al.* ^27^ reported high failure rate (68% out of 176 cases) among posts with a diameter less than or equal 1/3 of the root. Additionally, 76% out of 237 cases showed post failure with a post length equal or exceeding 50% of the root length. These conflicting results might be due to the difference in the type of the posts used as glass fiber posts were not used in Peutzfedlt ^27^ study. Moreover, the cases of the current study were treated under supervision of faculty members following the rubrics mentioned in the methods unlike the situation in Peutzfedlt ^27^ cases which were treated by unknown practitioners in private clinics.

The ideal amount of the remaining GP in the root after post preparation ranges between 3-5 mm, and this was found in 38.8% of the cases, while 61% of cases included more than 5 mm of remaining GP. Similarly, a study done by Mathar and Almutairi ^11^ found 28% of the assessed cases had 3-5 mm of remaining GP and 61% included more than 5 mm of remaining GP. The presence of higher percentage of cases including more than 5 mm GP remaining might be justified by the precautionary attitude of students in treating clinical cases. ^11^ Also, Al Maghrabi *et al.* ^16^ who used the same criteria in their cases, reported 3-5 mm remaining GP in 68% of cases done in King Abdulaziz university. ^16^ Another study performed in Jazan University found that 55.7% of the assessed cases were within the same range of remaining GP. ^5^

The strength of this study is inclusion of students of different educational levels starting from the initial clinical year (4 ^th^ year) to the internship year can provide comprehensive findings on the students’ performance in treating ETT with post and core. The high percentages of cases following the standard guidelines of post and core placement and cementation among variable teeth (incisors, premolars, and molars) as well as the lack of difference in clinical level between the students of different educational years might be justified by the presence of faculty supervision with each student in the educational clinics to limit the complications or errors that can happen in the patient’s mouth. Additionally, following explicit calibrated rubrics by students and faculty members in placing post and core restorations and assessing their quality might have positively affected the treatment outcome. Inclusion of different students of various dental educational levels presents generalized findings on the performance of students in the dental college of Imam Abdurrahman University. The variation of ETT assessed in the study can provide broad understanding to the errors encountered that might be relevant to the tooth type or arch type. This can improve the dental curriculum of undergraduate students in order to enhance their knowledge and psychomotor skills.

However, the current study had some limitations such as being restricted to Eastern province so the findings cannot be generalized to the broader dental population in Saudi Arabia. Also, it included undergraduate students with the exclusion of postgraduate students since the postgraduate endodontic and prosthodontics boards were recently introduced in the dental college, and the total number of enrolled students does not exceed ten students. Further studies are needed to compare the performance of undergraduate and postgraduate students. Moreover, assessment of post and core quality needs to be extended in other Saudi dental colleges to detect the weaknesses in students’ performance.

## Conclusions

The successful post and core treatment relies on multiple factors related to the success of endodontic treatment as well as the procedure and type of post and core placed. In the current study, dental students demonstrated satisfactory skill in post and core placement for endodontically treated teeth according to the guidelines. The majority of the dental students restored the endodontically treated teeth with glass fiber posts. No significant difference was noticed in the skills of dental students of different educational levels. The presence of supervision from faculty members in both endodontics and prosthodontics fields might be a fundamental cause of this high percentage of acceptable treatments in addition to treating cases of low difficulty according to AAE assessment form and guidelines.

## Data availability

Zenodo. Radiographical Assessment of Post and Core Placement Errors Encountered by Saudi Dental Students at Different Educational Levels, 10.5281/zenodo.8126789. ^28^

This project contains the following underlying data:

• Post data.xlsx (post and core data done by students).

Data are available under the terms of the Creative Commons Attribution 4.0 International license (CC-BY 4.0).
