# Supplementary material for: Radiographic assessment of dental post and core placement at different educational levels in an undergraduate student clinic: a 4-year retrospective study
Source: F1000Res. 2024 Mar 28;12:976. Originally published 2023 Aug 14. [Version 2] doi: 10.12688/f1000research.137421.2 (PMC11143398; doi:10.12688/f1000research.137421.2)
Supplement: Peer review history

# Reviewer report: Reviewer response for version 2

**DOI:** 10.5256/f1000research.163330.r282296

**Published:** 2024-05-31

**Authors:** Rania Moussa

**Affiliations:** 1 Department of Substitutive Dental Science, College of Dentistry, Taibah University, Madinah, Saudi Arabia

The article retrospectively studies the quality of endodontic treatment followed by post- and core treatment of patients attending a teaching institute and treated by students at different educational levels. The aim of the study is clear and scientifically relevant to the increased number of emerging dental colleges, where internal auditing and self-evaluation are essential to improving students' competence and preparing them for unsupervised real life.

 the methodology is well-written and detailed 

**The only comment that is missing in the methodology is that the grouping of the sample is not mentioned while reported in the results.**

**Please add the group details to the methodology**

 The results are well-documented and detailed.

 Discussion addressed the results very well and referred to relevant literature

Is the work clearly and accurately presented and does it cite the current literature?

Yes

If applicable, is the statistical analysis and its interpretation appropriate?

Yes

Are all the source data underlying the results available to ensure full reproducibility?

Yes

Is the study design appropriate and is the work technically sound?

Yes

Are the conclusions drawn adequately supported by the results?

Yes

Are sufficient details of methods and analysis provided to allow replication by others?

Yes

Reviewer Expertise:

A prosthodontist and faculty member

I confirm that I have read this submission and believe that I have an appropriate level of expertise to confirm that it is of an acceptable scientific standard.

# Reviewer report: Reviewer response for version 2

**DOI:** 10.5256/f1000research.163330.r260338

**Published:** 2024-04-19

**Authors:** Yuanita Lely Rachmawati

**Affiliations:** 1 Department of Community and Preventive Dentistry, Universitas Brawijaya, Malang, East Java, Indonesia

The authors have revised the manuscript as suggested. No further comments from me. Thank you.

Is the work clearly and accurately presented and does it cite the current literature?

Partly

If applicable, is the statistical analysis and its interpretation appropriate?

Partly

Are all the source data underlying the results available to ensure full reproducibility?

Partly

Is the study design appropriate and is the work technically sound?

Partly

Are the conclusions drawn adequately supported by the results?

No

Are sufficient details of methods and analysis provided to allow replication by others?

Partly

Reviewer Expertise:

Oral health literacy and OoL

I confirm that I have read this submission and believe that I have an appropriate level of expertise to confirm that it is of an acceptable scientific standard.

# Reviewer report: Reviewer response for version 1

**DOI:** 10.5256/f1000research.150581.r224915

**Published:** 2023-12-21

**Authors:** Yuanita Lely Rachmawati

**Affiliations:** 1 Department of Community and Preventive Dentistry, Universitas Brawijaya, Malang, East Java, Indonesia

**Comments:**

 I appreciate the hard work that all authors have put into this research.

 However, some details need to be added

**Keywords**

 Please check the selected words in the MeSH

**Methods**

 Please explain **in **more detail about sample size calculation.

 What Intraoral X-ray imaging unit was used and how the PA technique was taken

 “These PAs were assessed according to the following criteria by four

 trained investigators”. Please add information related to the investigator: who are they, how they got the training, and how to measure the agreement between them.

 Figure 1 looks blurry, the writing needs to be clarified.

**Results**

 At Methods section mentions “A total of 550 periapical radiographs” but in the Result section “The study included 502 students”. Please explain whether the analysis unit was PA or student, and the reasons why there are differences in numbers.

 One-way ANOVA was used in this study. One-way ANOVA is a statistical test that is used to measure differences in average data on numerical scale variables. The authors can add a table that informs the mean and SD values of the variables.

**Discussion**

 It is necessary to add references in the first paragraph.

 Please add the reference of the standard guidelines of post and core placement and cementation among variable teeth

**Conclusion**

 The content of the conclusion is focused on the research objectives and results

Is the work clearly and accurately presented and does it cite the current literature?

Partly

If applicable, is the statistical analysis and its interpretation appropriate?

Partly

Are all the source data underlying the results available to ensure full reproducibility?

Partly

Is the study design appropriate and is the work technically sound?

Partly

Are the conclusions drawn adequately supported by the results?

No

Are sufficient details of methods and analysis provided to allow replication by others?

Partly

Reviewer Expertise:

epidemiology

I confirm that I have read this submission and believe that I have an appropriate level of expertise to confirm that it is of an acceptable scientific standard, however I have significant reservations, as outlined above.

# Response

**Published:** 2024-03-02

**Authors:** Turki Alshehri

**Affiliations:** college of dentistry, Imam Abdulrahman Bin Faisal University, Dammam, Eastern Province, Saudi Arabia

Dear Editor and Reviewers,

 We would like to thank you for inviting us to resubmit our revised manuscript. We wanted to express our sincere appreciation for the time and effort you dedicated to reviewing our manuscript. Your thoughtful feedback and insights have been instrumental in refining the quality and impact of our work.

 We have carefully incorporated your suggestions into the revised manuscript, and we are confident that these revisions have significantly strengthened the overall content and presentation. Your thorough review has contributed to elevating the manuscript's potential to make a meaningful contribution to the prosthodontics community.

 In response to your invaluable comments, we have diligently revised the manuscript to address your concerns. Below, we have summarized the main points you raised and provided our responses, highlighted in yellow.

 Thank you for your time, consideration, and ongoing support.

 Sincerely

 Authors

 Comments:

 I appreciate the hard work that all authors have put into this research.

 However, some details need to be added.

 •    Keywords

 1.    Please check the selected words in the MeSH

 •    Response: Thank you for your valuable comment. Keywords were adjusted in the manuscript on page 15.

 •    Methods

 1.    Please explain in more detail about sample size calculation.

 •    Response: Thank you for your comment. The modifications were done in the methods section of the manuscript on page 3.

 2.    What Intraoral X-ray imaging unit was used and how the PA technique was taken?

 •    Response: Thank you for your raising this important point. The modification was done in the methods section of the manuscript on pages 4-5.

 3.    “These PAs were assessed according to the following criteria by four trained investigators”. Please add information related to the investigator: who are they, how they got the training, and how to measure the agreement between them.

 •    Response: Thank you for your important comment. The assessment method was clarified in the methods section of the manuscript on page 4.

 4.    Figure 1 looks blurry; the writing needs to be clarified.

 •    Response: Thank you for your valuable comment. Figure resolution was adjusted in the methods section on page 6.

 •    Results

 1.    At Methods section mentions “A total of 550 periapical radiographs” but in the Result section “The study included 502 students”. Please explain whether the analysis unit was PA or student, and the reasons why there are differences in numbers.

 •    Response: Thank you for your raising this important point. This difference in the total number mentioned in the results was due to the missing data of 48 cases, and this statement was added in the results section on page 8.

 2.    One-way ANOVA was used in this study. One-way ANOVA is a statistical test that is used to measure differences in average data on numerical scale variables. The authors can add a table that informs the mean and SD values of the variables.

 •    Response: Thank you for your valuable comment. Table 1 includes the requested information on page 8.

 •    Discussion

 1.    It is necessary to add references in the first paragraph.

 •    Response: Thank you for your valuable comment. References were added to the introduction and rearranged in the reference section page number 14 and 15.

 2.    Please add the reference of the standard guidelines of post and core placement and cementation among variable teeth

 •    Response: Thank you for your valuable comment. The guidelines used were added in Table 1 and the reference of previous studies (5, 9, 11, 15) where the guidelines were extracted and added in the methods section pages 5-7.

 •    Conclusion

 1.    The content of the conclusion is focused on the research objectives and results.

 •    Response: Thank you for your valuable comment. The conclusion was adjusted on page 13.

# Reviewer report: Reviewer response for version 1

**DOI:** 10.5256/f1000research.150581.r214866

**Published:** 2023-10-24

**Authors:** Manal Matoug-Elwerfelli

**Affiliations:** 1 College of Dental Medicine, QU Health, Qatar University, Doha, Doha, Qatar

**Comments:**

 I thank the authors for submitting this manuscript and appreciates the resources, time and effort invested by the authors in producing this manuscript. However, I highlight several issues that needs to be addressed. Additionally, the English language and grammar needs to be improved throughout the manuscript. I elaborate on the main issues below:

**Title**

 Suggested change in title; Radiographic assessment of dental post and core placement at different educational levels in an undergraduate student clinic: a 4?-year retrospective study

**Keywords**

 Please ensure keywords are suitable Mesh terms

**Abstract**

 To be edited taken the below comments into consideration

**Introduction**

 The introduction needs to be improved, with a better structure including clinical/educational relevance of study and gap of knowledge, hence why the study was conducted.

 The authors are advised to read classical references of dental posts such as;

- Fernandes A. S., Dessai G. S. Factors affecting the fracture resistance of post-core reconstructed teeth: a review. Int. J. Prosthodont. 14, (2001).
- Trope M., Maltz D. O., Tronstad L. Resistance to fracture of restored endodontically treated teeth. Dent. Traumatol. 1, 108-111 (1985).

The authors mention “Accordingly, these rubrics provide proper assessment of the students’ practice and skills level, and also helps in improving their ability to perform self-assessment for each specific dental procedure.” Please elaborate on the rubrics? Consider adding as a supplementary file.

**Methods**

 As a general comment, The experimental design needs to be explained in more detail.

 Please read for further guidance;

-
**https://doi.org/10.1111/j.1365-2591.2009.01679.x**

- https://doi.org/10.7717/peerj.13858
- https://doi.org/10.1016/j.jdent.2004.01.002

I have mentioned a few of the most important points that must be included;

 Any sample size calculation to determine power of the study? Please explain

  Please add a statement about patient consent was waived due to nature of study. Also please add statement about anonymous patient information status.

  Please correct; dammam To  Dammam 

 Can the authors mention was the post placement placed on primary (non-surgical) root canal treatment or re-treatment cases?

 How were cases selected for undergraduate students? Please discuss the necessity for preoperative use of the AAE case difficulty assessment form and similar tools, especially in selecting appropriate teeth for inexperienced operators. Which cases were deemed as too difficult for students, and should this strategy be revised, or recommendations made considering the results?

 Please add; average staff to student ratio during clinical supervision

 Please provide further details about how the radiographs were taken? Parallel technique? Digital or manually developed?

 Please provide further details about; post placement and to determine if any errors.

 Please provide further details about the investigators (These PAs were assessed according to the following criteria by four trained investigators). Are they students, specialists, consultants?  How many years of training?

 Were the investigators calibrated? This should be added..How was calibration performed?

 Please clearly state inclusion and exclusion criteria.

 Please clearly mention how root canal treatment and canal obturation was performed?

 The authors mention (the assessment of the quality of canal preparation (acceptable: outline of the preparation was following the canal contour and unacceptable: canal preparation did not follow the canal outline or ledge resulted during preparation) and the quality of PAs were added to the excel sheet where the followed evaluation criteria of PAs were extracted from a previous study criteria (REF 11). Based on what criteria was the post/core assessed? on what criteria was the quality of canal preparation assessed? Please provide a suitable reference. Also the reference for the criteria of PAs is unsuitable, please use international guideline criteria

**Results**

 The authors state, “There were no statistically significant differences between dental students at different clinical educational years regarding the quality of post placement according to the criteria mentioned in the method.” These results are generally non-logical, is the quality of dental post/core placement for a 4 ^th^ year dental student comparable to dental interns.

 Findings of the study need to be explained in more detail.

 Is the statistical analysis the most appropriate? Please consult a statistician and report the results in details

 All table to be edited, as per above comment. Please consult a statistician for improvements

**Discussion**

 Please add suitable reference to the first paragraph.

 The authors must mention the endodontic pre-clinical and clinical teaching and training that the students have been taught? Additionally, what year do they start endodontic treatment? Post placement?

 The discussion should consist of comments on the methodologies chosen and comparison with previous studies.

 The authors keep referring to standard guidelines, please further elaborate. Which international guidelines are the authors referring too?

 Lack of clinical data to further support radiographic assessment to determine success/survival should be added

 Please add strengths of study?

 Additionally limitations can be added, please add

**Conclusion**

 How was the below statement extracted from the results; “Dental students’ fear of perforations of narrow canals or furcation perforations pushed them to treat mainly premolars and incisors and to use narrower posts than the proper diameter. Therefore, students need to have more confidence in their qualities to improve their clinical skills.” ?? Please remove if not directly extracted from the study results….

 Please re-write the conclusion based on the results of the study

**References**

 Errors in references are noted, To be carefully edited

Is the work clearly and accurately presented and does it cite the current literature?

Partly

If applicable, is the statistical analysis and its interpretation appropriate?

Partly

Are all the source data underlying the results available to ensure full reproducibility?

No

Is the study design appropriate and is the work technically sound?

Partly

Are the conclusions drawn adequately supported by the results?

No

Are sufficient details of methods and analysis provided to allow replication by others?

No

Reviewer Expertise:

Restorative dentistry and Endodontics

I confirm that I have read this submission and believe that I have an appropriate level of expertise to state that I do not consider it to be of an acceptable scientific standard, for reasons outlined above.

## References

[1] : Factors affecting the fracture resistance of post-core reconstructed teeth: a review. *Int J Prosthodont* .2001;14(4) :355-63 11508092

[2] : Resistance to fracture of restored endodontically treated teeth. *Endod Dent Traumatol* .1985;1(3) : 10.1111/j.1600-9657.1985.tb00571.x 108-11 10.1111/j.1600-9657.1985.tb00571.x 3893998

[3] : Technical quality of root fillings performed by undergraduate students in Saudi Arabia. *Int Endod J* .2010;43(4) : 10.1111/j.1365-2591.2009.01679.x 292-300 10.1111/j.1365-2591.2009.01679.x 20487448

[4] : Radiographic assessment of endodontic mishaps in an undergraduate student clinic: a 2-year retrospective study. *PeerJ* .2022;10: 10.7717/peerj.13858 e13858 10.7717/peerj.13858 35945941 PMC9357366

[5] : Radiographic technical quality of root canal treatment performed by dental students at the Dental Teaching Center in Jordan. *J Dent* .2004;32(4) : 10.1016/j.jdent.2004.01.002 301-7 10.1016/j.jdent.2004.01.002 15053913

# Response

**Published:** 2024-03-02

**Authors:** Turki Alshehri

**Affiliations:** college of dentistry, Imam Abdulrahman Bin Faisal University, Dammam, Eastern Province, Saudi Arabia

Dear Editor and Reviewers,

 We would like to thank you for inviting us to resubmit our revised manuscript. We wanted to express our sincere appreciation for the time and effort you dedicated to reviewing our manuscript. Your thoughtful feedback and insights have been instrumental in refining the quality and impact of our work.

 We have carefully incorporated your suggestions into the revised manuscript, and we are confident that these revisions have significantly strengthened the overall content and presentation. Your thorough review has contributed to elevating the manuscript's potential to make a meaningful contribution to the prosthodontics community.

 In response to your invaluable comments, we have diligently revised the manuscript to address your concerns. Below, we have summarized the main points you raised and provided our responses, highlighted in yellow.

 Thank you for your time, consideration, and ongoing support.

 Sincerely

 Authors

 Comments:

 1.    I thank the authors for submitting this manuscript and appreciate the resources, time, and effort invested by the authors in producing this manuscript. However, I highlight several issues that need to be addressed. Additionally, the English language and grammar need to be improved throughout the manuscript. I elaborate on the main issues below:

 •    Title: Suggested change in title; Radiographic assessment of dental post and core placement at different educational levels in an undergraduate student clinic: a 4-year retrospective study

 •    Response: Thank you for your valuable comment. The title was adjusted in the manuscript page number 1 according to the reviewer’s suggestion.

 2.    Keywords: Please ensure keywords are suitable Mesh terms.

 •    Response: Thank you for your raising this comment. Key words were adjusted in the manuscript page number 15.

 3.    Abstract To be edited take the below comments into consideration

 4.    Introduction

 1.    The introduction needs to be improved, with a better structure including the clinical/educational relevance of the study and the gap of knowledge, hence why the study was conducted.

 The authors are advised to read classical references of dental posts such as;

 Fernandes A. S., Dessai G. S. Factors affecting the fracture resistance of post-core reconstructed teeth: a review. Int. J. Prosthodont. 14, (2001).

 Trope M., Maltz D. O., Tronstad L. Resistance to fracture of restored endodontically treated teeth. Dent. Traumatol. 1, 108-111 (1985).

 •    Response: Thank you for your valuable comment. The introduction section was modified as requested on page numbers 1,2.

 2.    The authors mention “Accordingly, these rubrics provide a proper assessment of the student’s practice and skills level and also helps in improving their ability to perform self-assessment for each specific dental procedure.” Please elaborate on the rubrics. Consider adding it as a supplementary file.

 •    Response: Thank you for your comment. The rubrics were added in the methods section. They were extracted from previously published articles (5, 9, 11, 15) and verified by the prosthodontics faculty department in the dental college. The rubrics were formulated to be comprehensive including assessment of the quality of radiogragraphs, quality of obturation as well as quality of post and core preparation and placement. These criteria are explained in the methods section on pages 4-7 as well as in Table 1 and Figure 1

 •    Methods

 5.    As a general comment, the experimental design needs to be explained in more detail.

 Please read for further guidance;

 https://doi.org/10.1111/j.1365-2591.2009.01679.x 

 https://doi.org/10.7717/peerj.13858 

 https://doi.org/10.1016/j.jdent.2004.01.002

 •    Response: Thank you for your valuable comment. Methods are explained in detail in page numbers 4-7.

 I have mentioned a few of the most important points that must be included.

 6.    Any sample size calculation to determine the power of the study? Please explain.

 •    Response: Thank you for your important comment. The sample size was clarified in the methods section page number 3.

 7.    Please add a statement about patient consent being waived due to the nature of the study. Also please add a statement about anonymous patient information status.

 •    Response: Thank you for your valuable comment. The statements were added in the methods section on page 4.

 8.    Please correct; dammam To Dammam 

 •    Response: The city name was corrected in the methods section of the manuscript on page 3.

 9.    Can the authors mention was the post placement placed on primary (non-surgical) root canal treatment or re-treatment cases?

 •    Response: Thank you for your valuable comment. The inclusion and exclusion criteria were clarified in the methods section on page 4.

 10.    How were cases selected for undergraduate students? Please discuss the necessity for preoperative use of the AAE case difficulty assessment form and similar tools, especially in selecting appropriate teeth for inexperienced operators. Which cases were deemed as too difficult for students, and should this strategy be revised, or recommendations made considering the results?

 •    Response: Thank you for your important comment. Guidelines reference was added in the methods section on page 4.

 11.    Please add, the average staff-to-student ratio during clinical supervision

 •    Response: Thank you for your valuable comment. The ratio was added in the methods section in the manuscript page number 5.

 12.    Please provide further details about how the radiographs were taken. Parallel technique? Digital or manually developed?

 •    Response: Thank you for your comment. The technique was added in the methods section of the manuscript on page 3.

 13.    Please provide further details about; post placement and to determine if any errors.

 •    Response: Thank you for your comment. The errors are explained in detail in the methods section page number 4-7.

 14.    Please provide further details about the investigators (These PAs were assessed according to the following criteria by four trained investigators). Are they students, specialists, consultants?  How many years of training?

 •    Response: Thank you for your valuable comment. Criteria were mentioned in the methods section on page 4.

 15.    Were the investigators calibrated? This should be added. How was calibration performed?

 •    Response: Thank you for your valuable comment. The calibration method was added in the methods section on page 4.

 16.    Please clearly state inclusion and exclusion criteria.

 •    Response: Thank you for your valuable comment. The criteria were clarified in the methods section on page number 4.

 17.    Please clearly mention how root canal treatment and canal obturation were performed.

 •    Response: Thank you for your valuable comment. The procedure was added in the methods section on page number 4.

 18.    The authors mention (the assessment of the quality of canal preparation (acceptable: outline of the preparation was following the canal contour and unacceptable: canal preparation did not follow the canal outline or ledge resulted during preparation) and the quality of PAs were added to the excel sheet where the followed evaluation criteria of PAs were extracted from a previous study criteria (REF 11). Based on what criteria was the post/core assessed? on what criteria was the quality of canal preparation assessed? Please provide a suitable reference. Also, the reference for the criteria of PAs is unsuitable, please use international guideline criteria

 •    Response: Thank you for your comment. References to the guidelines used to assess post and core treatment quality were added in the methods section pages 4-5.

 •    Results

 19.    The authors state, “There were no statistically significant differences between dental students at different clinical educational years regarding the quality of post-placement according to the criteria mentioned in the method.” These results are generally non-logical, is the quality of dental post/core placement for a 4th year dental student comparable to dental interns?

 •    Response: Thank you for your valuable comment. The quality of treatment was comparable because the treatment procedures were done under faculty supervision from both departments’ endodontics and prosthodontics. This might explain the lack of significant difference in the treatment quality of all dental years included in the study. This justification was mentioned in pages 12 and 13.

 20. The findings of the study need to be explained in more detail.

 •    Response: Thank you for your valuable comment. The findings were thoroughly explained in the discussion section on pages 11 and 12.

 21.    Is the statistical analysis the most appropriate? Please consult a statistician and report the results in detail.

 •    Response: Thank you for your comment. All comments were adjusted in the result section pages 8-10.

 22.    All tables to be edited, as per the above comment. Please consult a statistician for improvements

 •    Response: Thank you for your valuable comment. The result section was adjusted as recommended on pages 8-10.

 •    Discussion

 23.    Please add a suitable reference to the first paragraph.

 •    Response: Thank you for your important comment. References were added to the introduction on page 3.

 24.    The authors must mention the endodontic pre-clinical and clinical teaching and training that the students have been taught. Additionally, what year do they start endodontic treatment? Post placement?

 •    Response: Thank you for your valuable comment. All details were added in the methods section page number 11.

 25.    The discussion should consist of comments on the methodologies chosen and a comparison with previous studies. 

 •    Response: Thank you for your comment. The discussion was adjusted according to the suggestion.

 26.    The authors keep referring to standard guidelines, please further elaborate. Which international guidelines are the authors referring to?

 •    Response: Thank you for your valuable comment. The guidelines of post and core placement and endodontic case selection were added to the methods (Table 1 and Figure 1) on pages 4-7.

 27.    Lack of clinical data to further support radiographic assessment to determine success/survival should be added.

 •    Response: Thank you for your comment. The data were added in the methods section and compared with previous similar studies.

 28.    Please add the strengths of the study.

 •    Response: Thank you for your valuable comment. Strengths are available in the discussion section on page 13.

 29.    Additionally, limitations can be added, please add

 •    Response: Thank you for your valuable comment. Limitations are mentioned in the discussion section on page 13.

 •    Conclusion

 30.    How was the below statement extracted from the results; “Dental students’ fear of perforations of narrow canals or furcation perforations pushed them to treat mainly premolars and incisors and to use narrower posts than the proper diameter. Therefore, students need to have more confidence in their qualities to improve their clinical skills.”?? Please remove if not directly extracted from the study results….

 Please re-write the conclusion based on the results of the study.

 •    Response: Thank you for your comment. The conclusion was adjusted on page 13.

 •    References

 31.    Errors in references are noted, and to be carefully edited.

 •    Response: Thank you for your valuable comment. All references were adjusted according to journal guidelines in the reference section.
